# Characterisation of Mediterranean Grape Pomace Seed and Skin Extracts: Polyphenolic Content and Antioxidant Activity

**DOI:** 10.3390/molecules20022190

**Published:** 2015-01-29

**Authors:** Isabelle Ky, Pierre-Louis Teissedre

**Affiliations:** 1 Univ Bordeaux, ISVV, EA 4577 Œnologie, 210 Chemin de Leysotte, Villenave d’Ornon F-33140, France; 2 INRA, ISVV, USC 1366 Œnologie, 210 Chemin de Leysotte, Villenave d’Ornon F-33140, France

**Keywords:** grape pomaces, Mediterranean varieties, phenolic compounds, flavan-3-ols, anthocyanins, antioxidant activity

## Abstract

Grape pomace seeds and skins from different Mediterranean varieties (Grenache [GRE], Syrah [SYR], Carignan [CAR], Mourvèdre [MOU] and Alicante [ALI]) were extracted using water and water/ethanol 70% in order to develop edible extracts (an aqueous extract [EAQ] and a 70% hydro-alcoholic extract [EA70]) for potential use in nutraceutical or cosmetic formulations. In this study, global content (total polyphenols, total anthocyanins and total tannins), flavan-3-ols and anthocyanins were assessed using HPLC-UV-Fluo-MS^n^. In addition, extract potential was evaluated by four different assays: Oxygen Radical Absorbance Capacity (ORAC), Ferric Reducing Antioxidant Potential assay (FRAP), Trolox equivalent antioxidant capacity (TEAC) or ABTS assay and 2,2-diphenyl-1-picrylhydrazyl (DPPH) radical scavenging assay. As expected, seed pomace extracts contained higher amounts of polyphenols then skin pomace extracts. Indeed, seeds from Syrah contained a particularly important amount of total polyphenols and tannins in both type of extract (up to 215.84 ± 1.47 mg of gallic acid equivalent [GAE]/g dry weight (DW) and 455.42 ± 1.84 mg/g DW, respectively). These extracts also expressed the highest antioxidant potential with every test. For skins, the maximum total phenolic was found in Alicante EAQ (196.71 ± 0.37 mg GAE/g DW) and in Syrah EA70 (224.92 ± 0.18 mg GAE/g DW). Results obtained in this article constitute a useful tool for the pre-selection of grape pomace seed and skin extracts for nutraceutical purposes.

## 1. Introduction

Phenolic compounds are currently receiving much attention because of their beneficial health effects related to their ability to protect against oxidative cell damage when antioxidant and pro-oxidant imbalances occur. Indeed, reactive species play both a beneficial and toxic role and the balance between them has to be maintained [1]. To prevent an overload of free radicals and peroxides, aerobic organisms use a sophisticated defense system which operates in both the intra- and extracellular aqueous phases and in membranes. Antioxidant defense strategies are committed to prevent the oxidative attack in its early moments by the formation of priming radicals as well as during the initiation and chain propagation stages under stress condition or during aging processes, excessive levels of reactive species may interrupt regular processes. The exposure of tissues to oxidative stress could then generate a cascade of degenerative processes [2]. 

Clinical and nutritional epidemiological studies have shown an inverse correlation between the consumption of polyphenol-enriched diets and a reduced risk of cardiovascular diseases, along with their ensuing complications and related mortality [3,4,5]. Actually, polyphenolic compounds serve as reducing agents in many biological systems by donating hydrogen, quenching singlet oxygen, acting as chelators and by trapping free radicals. Moreover, these antioxidant activities can help to limit oxidation of nucleic acids, proteins, lipids, which may initiate degenerative diseases such as neuro-degenerative disease, cancer, heart disease, chronic inflammation, dermal disorders and aging [6,7,8]. 

*Vitis vinifera* grapes, one of the most cultivated fruit crops in the world with an annual production of ~64 million metric tons in 2010 [9], are known to be rich in polyphenols. Each year, the wine making industry produces a substantial amount of grape by-products called pomaces which account for about 20% of the weight of the grapes used to make wine [10,11]. The high polyphenol content of grapes and the far from complete extraction of grape polyphenols during vinification, which typically reaches only *ca.* 30%–40%, depending on grape varieties, vineyard location and technological parameters of wine making including destemming, crushing, maceration and pressing [12,13] make grape pomace potentially a very abundant and relatively inexpensive source of a wide range of polyphenols, including monomeric and oligomeric proanthocyanidins and a diversity of anthocyanin glycosides [14,15,16]. Significant efforts have been devoted over the past decade to explore the potential of using grape pomace to produce functional food ingredients, such as natural antioxidants for nutrition fortification and food preservation [17]. Other alternative potential commercial uses of grape pomaces that have been advocated include food colorings and ingredients [18,19,20], dietary fibers [10,21], phytochemical products [22] and dietary supplements for disease prevention [23]. Therefore, the aim of this study was to analyse the potential in this respect of grape by-products from important Rhône Valley red wine cultivars: Grenache, Syrah, Carignan, Mourvèdre and Alicante. Seeds and skins were extracted using water and water/ethanol 70% in order to develop two types of edible extracts: aqueous extracts (EAQ) and hydro-alcoholic 70% extracts (EA70). We reported herein the total polyphenol, total anthocyanin and total tannin contents as well as the determination and quantification of flavan-3-ols (monomers, dimers) and anthocyanins using HPLC with absorbance, fluorescence and mass detection. Moreover, the antioxidant capacity of pomace extracts was assessed using four antioxidant assays (ABTS**^∙^**^+^, DPPH, FRAP and ORAC). The data may contribute to the selection of suitable seed and skin pomace extracts for the development of antioxidant- and polyphenolic-rich nutraceuticals.

## 2. Results and Discussion

### 2.1. Total Phenol, Total Tannin and Total Anthocyanin Analysis of Grape Pomace Seed and Skin Extracts

Seeds of Grenache (1st location), Syrah and Carignan [GRE1, SYR1 and CAR] and skins from Grenache (2nd location), Syrah (two different locations), Carignan, Mourvèdre and Alicante [GRE2, SYR1, SYR2, CAR, MOU and ALI] were extracted using water and 70% hydro-alcoholic solution, thus giving two types of samples: aqueous samples (EAQ) and 70% hydro-alcoholic samples (EA70). Aqueous and 70% hydro-alcoholic extracts were characterized for their overall composition by total phenol content, total tannin and total anthocyanin analysis via Folin-Ciocalteu assay, acidic hydrolysis and SO_2_ bleaching procedure. Results are presented in Table 1 for seed extracts and Table 2 concerning skin extracts.

**Table 1:** Total phenol contents, total tannins, total anthocyanins and flavan-3-ol monomers, dimers and trimer characterisation in EAQ and EA70 grape pomace seed extracts.

	Seeds-EAQ			Seeds-EA70		
	GRE1 ^a^	SYR1 ^a^	CAR ^a^	GRE1 ^a^	SYR1 ^a^	CAR ^a^
**Total composition:**						
**TPC**	128.22 ± 0.37a	215.93 ± 1.17c	186.08 ± 0.28b	195.66 ± 1.06a	207.38 ± 2.15b	215.84 ± 1.47b
**Total tannins**	157.02 ± 0.56a	266.87 ± 2.62b	264.61 ± 2.39b	302.86 ± 4.85a	455.42 ± 1.84b	423.11 ± 15.13b
**Total anthocyanins**	3.98 ± 0.16a	10.55 ± 0.56b	11.35 ± 0.51b	12.17 ± 0.51a	38.67 ± 4.34b	57.34 ± 1.86b
**Proanthocyanidins composition:**						
**C**	2.07 ± 0.09a	5.12 ± 0.04b	2.27 ± 0.00a	3.60 ± 0.02a	8.60 ± 0.00c	5.28 ± 0.03b
**EC**	0.98 ± 0.04a	3.76 ± 0.03b	0.94 ± 0.00a	1.46 ± 0.00a	5.24 ± 0.00c	2.02 ± 0.06b
**Σ Monomers**	3.04 ± 0.09a	8.88 ± 0.00b	3.21 ± 0.00a	5.07 ± 0.01a	13.84 ± 0.00c	7.29 ± 0.02b
**B_1_**	1.01 ± 0.08a	2.94 ± 0.01b	0.87 ± 0.01a	1.68 ± 0.01a	3.53 ± 0.01c	3.06 ± 0.00b
**B_2_**	0.70 ± 0.01a	2.23 ± 0.02b	0.68 ± 0.00a	0.84 ± 0.00a	2.16 ± 0.02c	1.29 ± 0.00b
**B_3_**	0.28 ± 0.02a	0.86 ± 0.00b	0.25 ± 0.00a	0.45 ± 0.02a	0.87 ± 0.02c	0.58 ± 0.01b
**B_4_**	0.51 ± 0.01b	0.85 ± 0.04c	0.11 ± 0.00a	Nd	0.53 ± 0.01	Nd
**Σ Dimers**	2.50 ± 0.05b	6.870 ± 0.03c	1.90 ± 0.01a	2.97 ± 0.00a	7.10 ± 0.03c	4.92 ± 0.01b
**C_1_**	0.48 ± 0.01a	2.00 ± 0.06b	0.53± 0.01a	0.54 ± 0.00a	1.25 ± 0.03c	0.83 ± 0.01b

^a^ GRE1, Grenache; SYR1, Syrah; CAR, Carignan. In units of mg gallic acid equivalent (GAE)/g DW of seeds for TPC and mg/g DW of seeds for total tannins, total anthocyanins and the quantification of proanthocyanidins. Data are expressed as the mean of triplicate ± standard deviation. TPC, total phenol contents; C, (+)-Catechin; EC, (–)-Epicatechin; B_1_, B_2_; B_3_, B_4_, Procyanidin dimers B_1_, B_2_; B_3_, B_4_; C1, procyanidin trimers C_1_. Σ Monomers, sum of catechin and epicatechin; Σ Dimers, sum of B_1_, B_2_, B_3_ and B_4_; C_1_, trimer C_1_; Nd, Not determined. a, b, c; ANOVA was made to compare values obtain between varieties for the same compound. Same letters indicate no significant differences between the value (Tukey’s test, *p* < 0.05).

**Table 2:** Total phenol contents, total tannins, total anthocyanins and flavan-3-ol monomers, dimers and trimer characterisation in EAQ and EA70 grape pomace skin extracts.

	Skins-EAQ					
	GRE2 ^a^	SYR1 ^a^	SYR2 ^a^	CAR ^a^	MOU ^a^	ALI ^a^
**TPC**	109.72 ± 0.19c	146.50 ± 1.19e	71.88 ± 0.08a	120.83 ± 1.12d	102.27 ± 0.38b	196.71 ± 0.37f
**Total tannins**	112.28 ± 2.67b	156.63 ± 2.63c	86.36 ± 1.86a	161.61 ± 1.32c	104.79 ± 2.00b	221.4 ± 3.47d
**Total anthocyanins**	8.70 ± 0.01c	16.01 ± 0.01d	1.76 ± 0.01a	14.62 ± 0.75d	5.65 ± 0.01b	21.40 ± 0.20e
**Proanthocyanidins composition:**						
**C**	0.764 ± 0.003a	1.415 ± 0.012c	0.523 ± 0.057a	1.013 ± 0.003b	0.656 ± 0.011a	2.027 ± 0.127d
**EC**	0.285 ± 0.001a	1.043 ± 0.012b	0.370 ± 0.032a	0.352 ± 0.008a	0.377 ± 0.001a	1.368 ± 0.088c
**Σ Monomers**	1.050 ± 0.002ab	2.460 ± 0.020c	0.890 ± 0.060a	1.360 ± 0.010b	1.030 ± 0.010ab	3.400 ± 0.1500d
**B_1_**	0.621 ± 0.002ab	0.918 ± 0.008b	0.368 ± 0.003a	0.736 ± 0.007b	0.618 ± 0.002ab	0.908 ± 0.172b
**B_2_**	0.410 ± 0.002a	0.660 ± 0.006bc	0.363 ± 0.038a	0.433 ± 0.009a	0.568 ± 0.004b	0.771 ± 0.048c
**B_3_**	0.278 ± 0.003a	0.387 ± 0.007a	0.175 ± 0.006a	0.277 ± 0.010a	0.288 ± 0.007a	0.345 ± 0.115a
**B_4_**	Nd	Nd	Nd	Nd	Nd	0.317 ± 0.091
**Σ Dimers**	1.310 ± 0.002ab	1.960 ± 0.004bc	0.910 ± 0.030a	1.450 ± 0.020ab	1.470 ± 0.010ab	2.340 ± 0.300c
**C_1_**	0.469 ± 0.000ab	0.817 ± 0.012b	0.344 ± 0.082a	0.537 ± 0.002ab	0.547 ± 0.125ab	0.665 ± 0.146ab
	**Skins-EA70**					
	**GRE2 ^a^**	**SYR1 ^a^**	**SYR2 ^a^**	**CAR ^a^**	**MOU ^a^**	**ALI ^a^**
**TPC**	195.15 ± 0.28c	224.92 ± 0.18f	173.58 ± 0.08a	203.47 ± 0.83d	219.88 ± 0.18e	188.94 ± 0.69b
**Total tannins**	256.07 ± 3.65a	312.46 ± 10.77bc	250.17 ± 7.07a	345.34 ± 4.18c	268.6 ± 11.68ab	232.65 ± 3.14a
**Total anthocyanins**	53.66 ± 0.83a	86.68 ± 1.71b	45.38 ± 0.20a	88.44 ± 0.59b	46.64 ± 0.39a	54.41 ± 2.66a
**Proanthocyanidins composition:**						
**C**	1.420 ± 0.005a	2.287 ± 0.100b	2.094 ± 0.045b	1.440 ± 0.002a	1.522 ± 0.021a	5.084 ± 0.026c
**EC**	0.441 ± 0.003a	1.363 ± 0.008b	1.101 ± 0.195b	0.443 ± 0.003a	0.658 ± 0.001a	2.626 ± 0.005c
**Σ Monomers**	1.860 ± 0.001a	3.650 ± 0.080c	3.190 ± 0.110b	1.880 ± 0.003a	2.180 ± 0.020a	7.710 ± 0.020d
**B_1_**	0.915 ± 0.006a	1.266 ± 0.009b	1.190 ± 0.164ab	1.140 ± 0.013ab	1.150 ± 0.011ab	2.589 ± 0.004c
**B_2_**	0.396 ± 0.021a	0.635 ± 0.008c	0.602 ± 0.008bc	0.388 ± 0.021a	0.566 ± 0.001b	1.284 ± 0.001d
**B_3_**	0.297 ± 0.001b	0.292 ± 0.003b	0.360 ± 0.001c	0.265 ± 0.003a	0.305 ± 0.002b	0.603 ± 0.011d
**B_4_**	Nd	Nd	Nd	Nd	Nd	0.350 ± 0.000
**Σ Dimers**	1.610 ± 0.010a	2.190 ± 0.003c	2.150 ± 0.110c	1.790 ± 0.030ab	2.020 ± 0.010bc	4.830 ± 0.010d
**C_1_**	0.336 ± 0.002a	0.435 ± 0.001ab	0.662 ± 0.133b	0.307 ± 0.002a	0.371 ± 0.004a	0.629 ± 0.003b

^a^ GRE2, Grenache; SYR1 and SYR2, Syrah; CAR, Carignan; MOU, Mourvèdre, ALI, Alicante. In units of mg GAE/g DW of skins for TPC and mg/g DW of skins for total tannins, total anthocyanins and the quantification of proanthocyanidins. Data are expressed as the mean of triplicate ± standard deviation. TPC, total phenol contents; C, (+)-Catechin; EC, (–)-Epicatechin; B_1_, B_2_; B_3_, B_4_, Procyanidin dimers B_1_, B_2_; B_3_, B_4_; C1, procyanidin trimers C_1_. Σ Monomers, sum of catechin and epicatechin; Σ Dimers, sum of B_1_, B_2_, B_3_ and B_4_; C_1_, trimer C_1_; Nd, Not determined. a, b, c, d, e, f; ANOVA was made to compare values obtain between varieties for the same compound. Same letters indicate no significant differences between the value (Tukey’s test, *p* < 0.05).

Overall, the results showed that the use of a 70% hydro-alcoholic solution allowed a better extraction of phenolic compounds whether in seeds or in skins. Among the seed extracts, seeds from SYR1 and CAR were particularly rich in polyphenols, tannins and anthocyanins in EAQ and EA70 extracts (Table 1). SYR 1 (EA70) contained a higher tannins concentration, up to 455.42 mg/g DW, while CAR (EA70) has a higher quantity in total anthocyanins (57.34 mg/g DW). In both extracts, phenolic contents in GRE1 were low in comparison with other varieties. Our results are in accordance with several studies which have reported a lower amount of total phenol and anthocyanin contents in products derived from Grenache variety compared to other varieties such as Syrah, Mourvèdre and Carignan [24,25]. Indeed, Grenache cultivar is known to be used for rosé or fortified wines production and it is typically blended with other varieties. Moreover Grenache is also known to have thin skin with high ripeness level and high susceptibility to oxidation [26]. 

For both type of seed extracts, SYR1 was the richest, whether in monomers (8.88 mg/g DW in EAQ and 13.84 mg/g DW in EA70), in dimers (6.87 mg/g DW in EAQ and 7.10 mg/g DW in EA70) and trimer C_1_ (2.00 mg/g DW in EAQ and 1.25 mg/g DW in EA70) as opposed to GRE1 (Table 1). The latter had already appeared to contain low amount of polyphenols in previous total analysis. However, despite this low content, GRE1 still possessed an exploitable potential, especially when extracted with 70% hydro-alcoholic solution.

Concerning skins, results for EAQ extracts revealed that ALI contained the highest phenolic contents for the three tests combined (TPC: 196.71 mg GAE/g DW, total tannins: 221.40 mg/g DW and total anthocyanins: 21.40 mg/g DW) while in EA70, SYR1 skins were predominantly high in phenolic contents (total phenol contents: 224.92 mg GAE /g DW, total tannins: 312.46 mg/g DW and total anthocyanins: 86.68 mg/g DW) (Table 2). The poorest extract was SYR2, whether in EAQ or in EA70.

In greater detail, EAQ values ranged from 0.89 mg/g DW to 3.4 mg/g DW for the sum of monomers, from 0.91 mg/g DW to 2.34 mg/g DW for the sum of dimers and values from 1.88 mg/g DW to 7.71 mg/g DW and 1.61 mg/g DW to 4.83 mg/g DW were found in EA70 extracts, for the sum of monomers and dimers, respectively. ALI and SYR1 showed a higher content of flavan-3-ol monomers and dimers in both type of extract. Regarding the dimer B_4_ which could not be found in grape pomace skins except for those of Alicante varieties, a previous study has already reported this phenomenon in grape skins [27,28]. Among EAQ samples, SYR2 skin extracts was evidenced as having fewer amounts than other skin EAQ samples, but in EA70 it was GRE2 and CAR. The difference of polyphenolic content between SYR1 and SYR2 could be explained by the fact that SYR1 and SYR2 were derived from grapes from different parcels. Several studies have demonstrated the importance of climatic and geographical factors and cultural practices [29,30]. Moreover, this difference could also be explained by technical processes [12,13]. Actually, SYR1 and SYR2 grapes were used to make different wines and as a result the vinification process employed differed. For instance, in the case of SYR1, fermentation lasted 19 days whereas it lasted 22 day for SYR2. 

As it was already observed in seed extracts, EA70 were characterized by higher total phenol contents, total tannins and total anthocyanins. This result illustrated a better extraction by 70% alcoholic solution. Fournand *et al.* [31] reported that tannin extraction efficiency in a hydroalcoholic solution similar to wine was lower than 38%. Indeed only a small amount of tannins are released during fermentation and this resulted in a fermented pomace with high tannin contents and increased tannin extractibility. Ethanol can facilitate tissue dissolution and thus, liberate a greater amount of polyphenols. Actually, between EAQ and EA70, total polyphenols and total tannins rate were 1.5- to 2.5- and 1- to 3-fold respectively, higher in EA70. Furthermore, beside the solvent effect, previous studies by Vergara-Salinas *et al.* [32] have shown that fermented pomace could also facilitate the extraction of tannins compared to unfermented ones.

### 2.2. Anthocyanin Analysis of Grape Pomace Seed and Skin Extracts by HPLC-UV-MS^n^

In total, 18 anthocyanins were detected by HPLC-PDA-MS. Compounds were identified on the basis of their absorbance spectra, the retention times of commercially available standards, elution order, *m/z* of the positively charged molecular ion ([M]^+^) and on the MS^2^ fragmentation, according to previous reports. Table 3 and Table 4 summarize the anthocyanin contents of grape pomace seed and skin extracts in which total anthocyanins represents the sum of individual anthocyanins.

Inall studied varieties, the main compounds consisted of 3*-O-*monoglucosides ([M−162]^+^) of delphinidin (*m/z* 303), cyanidin (*m/z* 287), petunidin (*m/z* 317), peonidin (*m/z* 301) and malvidin (*m/z* 331) which accounted for 63% in EAQ, 64% in EA70 to 72% in EAQ, 70% in EA70 of the total anthocyanins content in seed and skin pomace extracts, respectively. 

**Table 3:** Anthocyanin characterisation in EAQ and EA70 grape pomace seed extracts.

	Seeds-EAQ			Seeds-EA70		
	GRE1 ^a^	SYR1 ^a^	CAR ^a^	GRE1 ^a^	SYR1 ^a^	CAR ^a^
**Dp-3-*O*-Glc**	0.03 ± 0.00a	0.08 ± 0.00b	0.40 ± 0.00c	0.19 ± 0.00a	0.31 ± 0.00b	3.11 ± 0.02c
**Cy-3-*O*-Glc**	0.02 ± 0.00a	0.02 ± 0.00a	0.05 ± 0.00b	0.09 ± 0.00b	0.05 ± 0.00a	0.23 ± 0.00c
**Pt-3-*O*-Glc**	0.05 ± 0.00a	0.14 ± 0.00b	0.43 ± 0.00c	0.33 ± 0.01a	0.58 ± 0.02b	3.18 ± 0.01c
**Pn-3-*O*-Glc**	0.11 ± 0.00a	0.12 ± 0.00b	0.15 ± 0.01c	0.56 ± 0.03a	0.47 ± 0.02a	1.11 ± 0.00b
**Mv-3-*O*-Glc**	0.39 ± 0.00a	0.93 ± 0.00b	1.48 ± 0.02c	2.36 ± 0.12a	3.57 ± 0.08b	10.52 ± 0.11c
**Mv-3-*O*-Glc-acetaldehyde (vitisin B)**	Nd	0.02 ± 0.00b	0.02 ± 0.00a	0.01 ± 0.00a	0.05 ± 0.00c	0.03 ± 0.00b
**Dp-3-*O*-(6"-*O*-acetyl)-Glc**	Nd	0.02 ± 0.00	Nd	Nd	0.09 ± 0.00b	0.06 ± 0.00a
**Dimer Mv-Cat**	0.01 ± 0.00a	0.03 ± 0.00b	Nd	0.04 ± 0.00a	0.05 ± 0.00b	0.06 ± 0.00c
**Mv-3-*O*-glc-pyruvate (vitisin A)**	0.02 ± 0.00a	0.04 ± 0.00b	0.04 ± 0.00b	0.12 ± 0.00b	0.07 ± 0.00a	0.14 ± 0.00c
**Dimer Mv-Cat**	0.002 ± 0.00a	0.07 ± 0.00c	0.05 ± 0.00b	Nd	0.13 ± 0.00a	0.17 ± 0.00b
**Dimer Mv-Cat**	0.01 ± 0.00a	0.03 ± 0.00c	0.02 ± 0.00b	0.10 ± 0.00a	0.13 ± 0.00b	0.16 ± 0.00c
**Pn-3-*O*-(6"-*O*-acetyl)-Glc**	0.01 ± 0.00a	0.07 ± 0.00b	Nd	0.07 ± 0.00a	0.32 ± 0.01c	0.12 ± 0.00b
**Mv-3-*O*-(6"-*O*-acetyl)-Glc**	0.02 ± 0.00a	0.32 ± 0.00c	0.09 ± 0.00b	0.06 ± 0.00a	0.9 ± 0.03c	0.31 ± 0.00b
**Dp-3-*O*-(6"-*O*-coumaroyl)-Glc**	0.005 ± 0.00a	0.06 ± 0.00b	0.10 ± 0.00c	0.05 ± 0.00a	0.18 ± 0.00b	0.58 ± 0.01c
**Mv-3-*O*-(6"-*O*-caffeoyl)-Glc**	0.01 ± 0.00a	0.04 ± 0.00c	0.02 ± 0.00b	0.08 ± 0.00a	0.11 ± 0.00b	0.33 ± 0.00c
**Cy-3-*O*-(6"-*O*-coumaroyl)-Glc**	0.01 ± 0.00a	0.02 ± 0.00b	0.02 ± 0.00b	0.07 ± 0.00a	0.07 ± 0.00a	0.11 ± 0.00b
**Pt-3-*O*-(6"-*O*-coumaroyl)-Glc**	0.01 ± 0.00a	0.11 ± 0.00b	0.11 ± 0.00b	0.08 ± 0.00a	0.35 ± 0.01b	0.66 ± 0.00c
**Mv-3-*O*-(6"-*O*-coumaroyl)-Glc**	0.06 ± 0.00a	1.14 ± 0.01c	0.65 ± 0.00b	0.80 ± 0.00a	2.85 ± 0.00b	4.51 ± 0.04c
**Total anthocyanins Glc**	**0.60 ± 0.00a**	**1.30 ± 0.00b**	**2.52 ± 0.03c**	**3.53 ± 0.16a**	**4.99 ± 0.12b**	**18.15 ± 0.09c**
**Total anthocyanins acetylated**	**0.03 ± 0.00a**	**0.41 ± 0.00c**	**0.09 ± 0.00b**	**0.13 ± 0.00a**	**1.30 ± 0.02c**	**0.49 ± 0.00b**
**Total anthocyanins coumaroylated**	**0.08 ± 0.00a**	**1.32 ± 0.01c**	**0.88 ± 0.00b**	**1.00 ± 0.00a**	**3.44 ± 0.01b**	**5.86 ± 0.02c**
**Total anthocyanins**	**0.76 ± 0.00a**	**3.26 ± 0.01b**	**3.63 ± 0.03c**	**5.00 ± 0.17a**	**10.28 ± 0.14b**	**25.38 ± 0.11c**

^a^ GRE1, Grenache; SYR1, Syrah; CAR, Carignan; Dp, Delphinidin; Cy, Cyanidin; Pt, Petunidin; Pn, Peonidin, Mv, Malvidin; Cat, Catechin; Glc, glucoside; Nd, not determined. Data are expressed as the mean of triplicate ± standard deviation as mg malvidin-3*-O-*glucoside equivalents/g DW of skins. a, b, c; ANOVA was made to compare values obtain between varieties for the same compound. Same letters indicate no significant differences between the value (Tukey’s test, *p* < 0.05).

**Table 4:** Anthocyanin characterisation in EAQ and EA70 grape pomace skin extracts.

	Skins-EAQ					
	GRE2 ^a^	SYR1 ^a^	SYR2 ^a^	CAR ^a^	MOU ^a^	ALI ^a^
**Dp-3-*O*-glc**	0.24 ± 0.01d	0.15 ± 0.00b	0.01 ± 0.00a	0.53 ± 0.00f	0.20 ± 0.00c	0.31 ± 0.00e
**Cy-3-*O*-glc**	0.09 ± 0.00d	0.01 ± 0.00a	Nd	0.05 ± 0.00b	0.07 ± 0.00c	0.11 ± 0.00e
**Pt-3-*O*-glc**	0.33 ± 0.01c	0.25 ± 0.01b	0.03 ± 0.00a	0.54 ± 0.00e	0.30 ± 0.00c	0.47 ± 0.02d
**Pn-3-*O*-glc**	0.35 ± 0.01c	0.2 ± 0.01b	0.02 ± 0.00a	0.19 ± 0.00b	0.22 ± 0.00b	1.49 ± 0.03d
**Mv-3-*O*-glc**	1.78 ± 0.05d	1.54 ± 0.05c	0.11 ± 0.00a	1.74 ± 0.02d	1.01 ± 0.01b	3.14 ± 0.03e
**Mv-3-*O*-glc-acetaldehyde (vitisin B)**	0.01 ± 0.00a	0.02 ± 0.00b	Nd	0.02 ± 0.00b	0.01 ± 0.00a	Nd
**Dp-3-*O*-(6"-*O*-acetyl) glc**	Nd	0.05 ± 0.00b	Nd	0.02 ± 0.00a	Nd	Nd
**Dimer Mv-Cat**	0.01 ± 0.00a	0.03 ± 0.00b	Nd	Nd	0.01 ± 0.00a	0.03 ± 0.00b
**Mv-3-*O*-glc-pyruvate (vitisin A)**	0.05 ± 0.00c	0.04 ± 0.00bc	0.02 ± 0.00a	0.04 ± 0.00b	0.06 ± 0.00d	0.10 ± 0.00e
**Dimer Mv-Cat**	Nd	0.08 ± 0.00c	Nd	0.05 ± 0.00b	0.01 ± 0.00a	0.05 ± 0.00b
**Dimer Mv-Cat**	0.01 ± 0.00bc	0.03 ± 0.00d	0.01 ± 0.00c	0.01 ± 0.00a	0.01 ± 0.00ab	0.03 ± 0.00d
**Pn-3-*O*-(6"-*O*-acetyl)-glc**	0.02 ± 0.00c	0.10 ± 0.00e	0.01 ± 0.00a	0.02 ± 0.00b	0.01 ± 0.00ab	0.09 ± 0.00d
**Mv-3-*O*-(6"-*O*-acetyl)-glc**	Nd	0.52 ± 0.00e	0.02 ± 0.00a	0.07 ± 0.00c	0.04 ± 0.00b	0.19 ± 0.00d
**Dp-3-*O*-(6"-*O*-coumaroyl)-glc**	Nd	0.09 ± 0.00c	Nd	0.13 ± 0.00d	0.02 ± 0.00a	0.06 ± 0.00b
**Mv-3-*O*-(6"-*O*-caffeoyl)-glc**	0.02 ± 0.00a	0.05 ± 0.00c	Nd	0.03 ± 0.00b	0.02 ± 0.00a	0.05 ± 0.00c
**Cy-3-*O*-(6"-*O*-coumaroyl)-glc**	0.01 ± 0.00a	0.02 ± 0.00b	Nd	0.03 ± 0.00c	0.03 ± 0.00d	0.03 ± 0.00c
**Pt-3-*O*-(6"-*O*-coumaroyl)-glc**	0.04 ± 0.00b	0.16 ± 0.00e	Nd	0.12 ± 0.00d	0.03 ± 0.00a	0.07 ± 0.00c
**Mv-3-*O*-(6"-*O*-coumaroyl)-glc**	0.22 ± 0.00c	1.59 ± 0.00f	0.01 ± 0.00a	0.68 ± 0.01d	0.15 ± 0.00b	1.11 ± 0.01e
**Total anthocyanins glc**	**2.8 ± 0.07d**	**2.15 ± 0.06c**	**0.17 ± 0.00a**	**3.05 ± 0.02e**	**1.8 ± 0.01b**	**5.52 ± 0.08f**
**Total anthocyanins acetylated**	**0.02 ± 0.00a**	**0.67 ± 0.00e**	**0.03 ± 0.00a**	**0.11 ± 0.00c**	**0.05 ± 0.00b**	**0.27 ± 0.00d**
**Total anthocyanins coumaroylated**	**0.27 ± 0.00c**	**1.86 ± 0.00f**	**0.01 ± 0.00a**	**0.96 ± 0.01d**	**0.24 ± 0.00b**	**1.27 ± 0.01e**
**Total anthocyanins**	**3.19 ± 0.08c**	**4.92 ± 0.06e**	**0.24 ± 0.00a**	**4.25 ± 0.03d**	**2.2 ± 0.01b**	**7.32 ± 0.08f**
	**Skins-EA70**					
	**GRE2 ^a^**	**SYR1 ^a^**	**SYR2 ^a^**	**CAR ^a^**	**MOU ^a^**	**ALI ^a^**
**Dp-3-*O*-glc**	1.43 ± 0.00b	0.78 ± 0.00a	0.97 ± 0.00a	5.35 ± 0.21d	2.35 ± 0.00c	1.06 ± 0.01a
**Cy-3-*O*-glc**	0.34 ± 0.02d	0.05 ± 0.00a	0.12 ± 0.00b	0.39 ± 0.01e	0.52 ± 0.00f	0.24 ± 0.00c
**Pt-3-*O*-glc**	2.05 ± 0.08b	1.29 ± 0.00a	1.53 ± 0.00a	5.04 ± 0.21d	3.38 ± 0.02c	1.65 ± 0.03a
**Pn-3-*O*-glc**	1.91 ± 0.02c	0.87 ± 0.01a	0.94 ± 0.02a	1.71 ± 0.03b	2.00 ± 0.01d	5.32 ± 0.01e
**Mv-3-*O*-glc**	10.96 ± 0.22c	7.59 ± 0.04b	6.76 ± 0.09a	14.82 ± 0.42d	10.55 ± 0.03c	11.18 ± 0.05c
**Mv-3-*O*-glc-acetaldehyde (vitisin B)**	0.03 ± 0.00b	0.06 ± 0.00e	0.07 ± 0.00f	0.05 ± 0.00d	0.04 ± 0.00c	0.03 ± 0.00a
**Dp-3-*O*-(6"-*O*-acetyl) glc**	0.04 ± 0.00a	0.16 ± 0.00e	0.13 ± 0.00d	0.09 ± 0.00c	0.05 ± 0.00ab	0.05 ± 0.00b
**Dimer Mv-Cat**	0.05 ± 0.00a	0.07 ± 0.00c	0.11 ± 0.00e	Nd	0.10 ± 0.00d	0.06 ± 0.00b
**Mv-3-*O*-glc-pyruvate (vitisin A)**	0.22 ± 0.01c	0.17 ± 0.01a	0.39 ± 0.00e	0.18 ± 0.01a	0.32 ± 0.00d	0.20 ± 0.00b
**Dimer Mv-Cat**	0.12 ± 0.00b	0.27 ± 0.01e	0.21 ± 0.00d	0.16 ± 0.00c	0.07 ± 0.00a	0.14 ± 0.00b
**Dimer Mv-Cat**	0.10 ± 0.00b	0.11 ± 0.00c	0.21 ± 0.01d	0.10 ± 0.00b	0.08 ± 0.00a	0.23 ± 0.00e
**Pn-3-*O*-(6"-*O*-acetyl)-glc**	0.17 ± 0.00a	0.52 ± 0.01c	0.39 ± 0.01bc	0.10 ± 0.00a	0.19 ± 0.00ab	0.52 ± 0.14c
**Mv-3-*O*-(6"-*O*-acetyl)-glc**	0.46 ± 0.01a	2.11 ± 0.05d	1.06 ± 0.00c	0.41 ± 0.02a	0.39 ± 0.00a	0.61 ± 0.00b
**Dp-3-*O*-(6"-*O*-coumaroyl)-glc**	0.25 ± 0.01b	0.12 ± 0.00a	0.35 ± 0.01e	0.75 ± 0.00f	0.30 ± 0.00d	0.27 ± 0.00c
**Mv-3-*O*-(6"-*O*-caffeoyl)-glc**	0.34 ± 0.01d	0.15 ± 0.00a	0.56 ± 0.00e	0.24 ± 0.00b	0.32 ± 0.00c	0.25 ± 0.00b
**Cy-3-*O*-(6"-*O*-coumaroyl)-glc**	0.13 ± 0.00b	0.05 ± 0.00a	0.17 ± 0.01d	0.13 ± 0.00b	0.52 ± 0.00e	0.14 ± 0.00c
**Pt-3-*O*-(6"-*O*-coumaroyl)-glc**	0.36 ± 0.00b	0.20 ± 0.00a	0.51 ± 0.01c	0.82 ± 0.01f	0.65 ± 0.00e	0.55 ± 0.00d
**Mv-3-*O*-(6"-*O*-coumaroyl)-glc**	3.09 ± 0.02b	1.53 ± 0.00a	4.12 ± 0.00e	3.80 ± 0.03d	3.24 ± 0.00c	6.25 ± 0.04f
**Total anthocyanins glc**	**16.68 ± 0.34b**	**10.59 ± 0.05a**	**10.33 ± 0.10a**	**27.3 ± 0.88d**	**18.79 ± 0.02c**	**19.45 ± 0.08c**
**Total anthocyanins acetylated**	**0.67 ± 0.01a**	**2.79 ± 0.04d**	**1.59 ± 0.01c**	**0.60 ± 0.02a**	**0.62 ± 0.00a**	**1.18 ± 0.14b**
**Total anthocyanins coumaroylated**	**3.82 ± 0.01b**	**1.89 ± 0.00a**	**5.14 ± 0.01d**	**5.494 ± 0.04e**	**4.71 ± 0.00c**	**7.21 ± 0.04f**
**Total anthocyanins**	**22.03 ± 0.35c**	**16.10 ± 0.10a**	**18.60 ± 0.11b**	**34.11 ± 0.95f**	**25.06 ± 0.01d**	**28.74 ± 0.01e**

^a^ GRE2. Grenache; SYR1 and SYR2. Syrah; CAR. Carignan; MOU. Mourvèdre; ALI. Alicante; Dp, Delphinidin; Cy, Cyanidin; Pt, Petunidin; Pn, Peonidin, Mv, Malvidin, Cat, Catechin; Glc, glucoside; Nd. not determined. Data are expressed as the mean of triplicate ± standard deviation as mg malvidin-3*-O-*glucoside equivalents/g DW of skins. a, b, c, d, e, f; ANOVA was made to compare values obtain between varieties for the same compound. Same letters indicate no significant differences between the value (Tukey’s test, *p* < 0.05).

Other compounds were largely represented by 3*-O-*(6"*-O-*coumaroyl) glucoside anthocyanins ([M−308] ^+^) followed by the 3*-O-*(6"*-O-*acetyl) glucoside one ([M−204]^+^) (Table 3 and Table 4). These results are in agreement with previously reported data which illustrated the predominantly monoglucoside character of *V. vinifera* species [33,34,35]. Besides, malvidin-3*-O-*glucoside and its derivatives, *p*-coumaroyl derivatives, petunidin-3*-O-*glucoside and peonidin-3*-O-*glucoside were the major compounds. Malvidin-3*-O-*glucoside alone accounted for 30% in seeds to 40% in skins, whereas the minor compound cyanidin-3*-O-*glucoside represented no more than 2% of the total anthocyanins.

In seed pomace extracts, an appreciable amount of anthocyanins still remained. This is due, in the first instance, to the contact between seeds and skins throughout the winemaking process, in particular during the pressing and maceration. Moreover, despite the separation of skins from seeds, the operation was not complete and some skins residues remained.

In the aqueous extracts, total anthocyanin contents ranged from 0.76 ± 0.001 mg/g DW to 3.63 ± 0.03 mg/g DW in GRE1 and CAR, respectively. CAR samples possessed the highest level of 3*-O-*glucoside anthocyanins (2.52 ± 0.03 mg/g DW) while SYR1 contained more acetylated and coumaroylated anthocyanins (0.41 ± 0.001 mg/g DW and 1.32 ± 0.01 mg/g DW respectively). Extracts of these two varieties contained four times more anthocyanins than Grenache, which was composed of only 0.76 ± 0.001 mg/g DW of total anthocyanins (Table 3).

Regarding the 70% hydro-alcoholic extract, overall, the level of anthocyanins was higher: 6.6-, 3.2- and 7-fold more anthocyanins were extracted from GRE1, SYR1 and CAR, respectively, than with the aqueous extraction method. Total anthocyanin levels ranged from 5.00 ± 0.17 mg/g DW in GRE1 to 25.38 ± 0.11 mg/g DW in CAR, which also possessed the highest levels of 3*-O-*glucosides and 3*-O-*(6"*-O-*coumaroyl) glucoside anthocyanins (18.15 ± 0.09 mg/g DW and 5.86 ± 0.02 mg/g DW respectively). Besides, an important amount of 3*-O-*(6"*-O-*acetyl) glucoside (1.3 ± 0.02 mg/g DW) was founded in SYR1 grape pomace seeds. Among the three studied varieties, the CAR variety proved to be a promising source of anthocyanins, especially in the EA70 extract compared to EAQ and the two other varieties. Data concerning Grenache are in good agreement with those obtained in total analysis (Table 1) and other comparative studies [24,25].

As expected, anthocyanin levels in skin pomace extracts were higher than those in seeds and the predominant compound was malvidin-3*-O-*glucoside, followed by petudinin-3*-O-*glucoside and peonidin-3*-O-*glucoside (Table 4). Previous studies showed that anthocyanins are extracted mainly in the aqueous phase during maceration prior to fermentation and at the beginning of alcoholic fermentation. Even though up to 77% of anthocyanins could be released in this process [31], a surprisingly large amount of anthocyanins still remained in grape pomace skins after the vinification process.

In aqueous extracts, the total anthocyanins value ranged from 0.24 ± 0.001 mg/g DW in SYR2 to 7.32 ± 0.08 mg/g DW in ALI. SYR1 possessed appreciable levels especially 3*-O-*(6"*-O-*acetyl) glucoside and 3*-O-*(6"*-O-*coumaroyl) glucoside anthocyanins. Concerning the 70% hydro-alcoholic extracts, levels ranged from 16.1 ± 0.1 mg/g DW in SYR1 to 34.11 ± 0.95 mg/g DW in CAR. Moreover, CAR, ALI and MOU were the varieties which possessed the highest amounts of anthocyanin 3*-O-*glucosides. Regarding the 3*-O-*(6"*-O-*acetyl) glucoside anthocyanins, the two Syrah (SYR1 and SYR2) retained the highest amounts reaching 2.79 ± 0.04 mg/g DW and 1.59 ± 0.01 mg/g DW, respectively. The 3*-O-*(6"*-O-*coumaroyl) glucoside anthocyanins were predominant in ALI, CAR and SYR2 (Table 4).

Considering the difference between the two extraction methods, the extraction yield was superior in 70% hydro-alcoholic extracts with the amounts, depending on the variety, varying from 3- to 77-fold higher. Surprisingly, in SYR2, 77 times more total anthocyanins were extracted. The level of 3*-O-*(6"*-O-*acetyl) glucoside and 3*-O-*(6"*-O-*coumaroyl) glucoside anthocyanins reached up to 1.59 ± 0.01 mg/g DW and 5.14 ± 0.01 mg/g DW, respectively. However, the two different methods of extraction did not strictly increase or decrease the ratio of 3*-O-*glucoside, 3*-O-*(6"*-O-*acetyl) glucoside and 3*-O-*(6"*-O-*coumaroyl) glucoside in grape skin and seed pomace extracts (Table 3 and Table 4). In some varieties, using 70% hydro-alcoholic extraction increased the relative amount of 3*-O-*(6"*-O-*coumaroyl) glucoside obtained, but in other instances, it decreased the ratio. For instance, GRE1 skin pomace extract comprised 9% coumaroylated anthocyanins in EAQ which increased to 17% when using 70% alcohol whereas in SYR1, 38% were extracted in EAQ and the ratio decreased to 12% in EA70. Overall, the data showed that the two different extraction methods did not substantially affect the ratio of 3*-O-*glucoside, 3*-O-*(6"*-O-*acetyl) glucoside and 3*-O-*(6"*-O-*coumaroyl) glucoside anthocyanins. Actually, this could be due to the structural differences between these compounds and their association with other constituents such as the adsorption on solids (yeast, pomace) or even the modifications in their structure (formation of tannin-anthocyanin complexes) [36]. Extraction with 70% of ethanol has nevertheless been shown to improve the recovery of anthocyanins from grape by-products. This result was in accordance with several works confirming that anthocyanin yields could be improved by using high ethanol concentration solvents. Cacae *et al.* [37] reported that extraction of anthocyanins from black currants using aqueous ethanol increased with ethanol concentration up to 60%. Another study by Lapornik *et al.* [38] showed that grape marc extracted with ethanol 70% had higher absolute values of anthocyanins than those extracted with water. Moreover, the anthocyanin extraction yield could also be enhanced by improving the extraction method as already shown by Howard *et al.* [39]. The authors studied anthocyanin contents in strawberry puree and demonstrated that strict oxygen exclusion during processing (*i.e.*, under carbon dioxide or nitrogen) could prevent oxidative reactions.

Among the studied varieties, CAR and ALI were shown to be a rich source of anthocyanins, especially with the 70% hydro-alcoholic extract. The SYR2 also contained substantial amounts of acetylated and coumaroylated anthocyanins in EA70.

### 2.3. Antioxidant Activities Evaluation of Grape Pomace Seed and Skin Extracts

The antioxidant potential was determined in order to select the most active grape pomace seeds and skins among the studied varieties. The assessment of antioxidant capacity has been the subject of extensive studies and arguments over the past decade. The choice of assay method is often based on speed, simplicity, ease of use and instrumentation availability. Generally, antioxidant measurements can be related either to the capacity of extracts to directly transfer hydrogen to a radical (DPPH or ABTS), to donate electrons (FRAP) or to act as competitors for peroxy radicals (ORAC test) [40]. Thus, the antioxidant capacity of each extract cannot be determined by a single method. More than one type of measurement needs to be performed to take into account the various mode of action of antioxidants [41,42]. In that context, in this work the free radical scavenging potential was evaluated by three spectrophotometric tests: the FRAP, ABTS^•+^ and DPPH and a spectrofluorimetric test, the ORAC test.

Concerning seed extracts, the four antioxidant analytical techniques gave the same classification both for EAQ and EA70. The highest antioxidant activities were found in SYR1 for both types of extracts. Results were correlated with previous analysis which evidenced SYR1 as having a substantial amount of flavan-3-ols, procyanidins and anthocyanins. GRE1 extract presented a low antioxidant activity as a consequence of its low phenolic contents (ORAC: 1466.4 µM TE/g DW; FRAP: 0.63 mM Fe^2+^/g DW, ABTS: 1203.2 µM TE/g DW and DPPH: 410.8 TE/g DW in EAQ and ORAC: 1926.7 µM TE/g DW; FRAP: 1.28 mM Fe^2+^/g DW, ABTS: 2813.1 µM TE/g DW and DPPH: 1277.6 TE/g DW in EA70). Antioxidant activities of EAQ and EA70 grape pomace seed extracts were showed in Table 5.

**Table 5:** Antioxidant activity characterisation in EAQ and EA70 grape pomace seed extracts.

	Seeds-EAQ			Seeds-EA70		
	GRE1 ^a^	SYR1 ^a^	CAR ^a^	GRE1 ^a^	SYR1 ^a^	CAR ^a^
**ORAC ^b^**	1466.39 ± 29.58a	2230.69 ± 101.74b	2058.58 ± 85.11b	1926.73 ± 108.55a	2613.98 ± 150.86a	2332.90 ± 91.94a
**FRAP ^b^**	0.63 ± 0.02a	1.33 ± 0.08c	1.06 ± 0.08b	1.28 ± 0.01a	1.45 ± 0.16a	1.20 ± 0.06a
**ABTS ^b^**	1203.20 ± 24.09a	2432.62 ± 55.95c	1948.75 ± 61.10b	2813.15 ± 89.95a	3601.20 ± 88.59b	3495.58 ± 66.40b
**DPPH ^b^**	410.79 ± 43.30a	1037.12 ± 64.04b	1050.59 ± 30.11b	1277.59 ± 54.69a	1685.87 ± 130.65b	1536.77 ± 38.92b

^a^ GRE1, Grenache; SYR1, Syrah; CAR, Carignan. Data are expressed as the mean of triplicate ± SD. ^b^ ORAC, ABTS and DPPH are expressed as µmol Trolox/g DW and FRAP as mmol Fe^2+^/g DW. a, b, c; ANOVA was made to compare values obtain between varieties for the same test. Same letters indicate no significant differences between the value (Tukey’s test, *p* < 0.05).

In skins, results obtained by the different antioxidant analyses were more disparate, especially in EA70 extracts (Table 6). In aqueous extracts, the highest antioxidant activity was found in SYR1 and ALI. This observation was observed with every test and correlated well with previous results evidencing these extracts as containing high phenolic content. In EA70, different antioxidant tests did not give the same extract classification. Despite this fact, SYR1 skin extract was classified as being the first or second extract showing the highest antioxidant capacity in the four tests (ORAC: 1912.6 µM TE/g DW; FRAP: 1.52 mM Fe^2+^/g DW, ABTS: 2614.5 µM TE/g DW and DPPH: 1391.7 TE/g DW).

Regression analyses (correlation coefficient R^2^) were attempted in order to correlate the results obtained with different methods. The best correlations with total phenolic contents were obtained with EAQ extracts both for seed and skin extracts: from R^2^ = 0.87 for DPPH to R^2^ = 0.99 for FRAP and from R^2^ = 0.79 for DPPH to R^2^ = 0.97 for ABTS in seed and skin extracts, respectively. Weaker correlations from R^2^ = 0.43 for ORAC to R^2^ = 0.72 for ABTS in seeds and from R^2^ = 0.34 for FRAP to R^2^ = 0.63 for ABTS in skins were observed in EA70 extracts. Positive correlations between TPC and antiradical activity using similar tests on grape seed samples and various plant samples have also been observed by other investigators [43,44,45]. Furthermore, this study demonstrated that correlations between grape pomace contents and antioxidant levels were higher with total values than with the specific compound concentrations quantified by HPLC. As noted in a recent publication [46], our result illustrated that antioxidant activity is more related to the total constituent levels than to the concentration of any individual compound, despite the fact that some compounds may contribute more than the others.

**Table 6:** Antioxidant activity characterisation in EAQ and EA70 grape pomace skin extracts.

	Skins-EAQ					
	GRE2 ^a^	SYR1 ^a^	SYR2 ^a^	CAR ^a^	MOU ^a^	ALI ^a^
**ORAC ^b^**	1190.70 ± 183.58ab	1345.94 ± 19.15ab	1065.98 ± 84.21a	1077.76 ± 60.16a	1033.76 ± 77.61a	1714.62 ± 14.77b
**FRAP ^b^**	0.56 ± 0.01c	0.88 ± 0.01e	0.14 ± 0.02a	0.67 ± 0.02d	0.32 ± 0.01b	1.13 ± 0.00f
**ABTS ^b^**	934.12 ± 11.9b	1427.98 ± 54.80c	668.30 ± 29.99a	1048.83 ± 101.57b	965.59 ± 16.63b	1760.08 ± 91.03d
**DPPH ^b^**	99.45 ± 10.82a	690.29 ± 147.01bc	263.85 ± 71.54ab	591.01 ± 85.59abc	279.43 ± 61.65ab	1057.12 ± 45.22c
	**Skins-EA70**					
	**GRE2 ^a^**	**SYR1 ^a^**	**SYR2 ^a^**	**CAR ^a^**	**MOU ^a^**	**ALI ^a^**
**ORAC ^b^**	1828.26 ± 40.37bc	1912.56 ± 6.09bc	1701.83 ± 88.34bc	1238.38 ± 11.09a	2070.03 ± 60.64c	1628.45 ± 82.58b
**FRAP ^b^**	1.32 ± 0.03c	1.52 ± 0.05d	0.94 ± 0.03a	1.34 ± 0.03c	1.03 ± 0.02ab	1.13 ± 0.01b
**ABTS ^b^**	2612.08 ± 130.93a	2614.5 ± 10.42a	2010.64 ± 146.96a	2555.92 ± 146.04a	2674.84 ± 187.30a	1923.37 ± 87.01a
**DPPH ^b^**	876.96 ± 74.32a	1391.69 ± 37.24bc	1164.91 ± 55.55ab	1075.39 ± 46.16ab	833.28 ± 26.37a	1749.31 ± 112.65c

^a^ GRE2, Grenache; SYR1 and SYR2, Syrah; CAR, Carignan; MOU, Mourvèdre; ALI, Alicante. Data are expressed as the mean of triplicate ± SD. ^b^ ORAC, ABTS and DPPH are expressed as µmol Trolox/g DW and FRAP as mmol Fe^2+^/g DW. a, b, c, d, e, f; ANOVA was made to compare values obtain between varieties for the same test. Same letters indicate no significant differences between the value (Tukey’s test, *p* < 0.05).

Actually, the antioxidant activities of EAQ and EA70 followed the same trend as the phenol content of the extracts. EA70 extracts exhibited higher potential and proved to be more effective than EAQ extracts. However, due to the great diversity of polyphenols, the structure-activity relationship and bioavailability, the therapeutic efficacy of the antioxidants differs extensively [41,42,47]. Many reports still showed inconsistent and conflicting results using different approaches for the assessment of antioxidant capacity, making difficult the development of a universal method by which antioxidant activity can be measured accurately and quantitatively. Because of bioavailability, metabolism, biotransformation and chemical reactivity, *in vitro* capacity cannot be simply extrapolated [48]. Therefore, in order to evaluate the health effects of these extracts, *in vivo* experiments need to be performed and the effects of antioxidant may be evaluated using appropriate biomarkers in biological fluids and tissues. Nevertheless, *in vitro* antioxidant activity assays could be used as a pre-selection tool for the choice of grape pomace seed and skin extracts with high potential. These experiments evidenced seeds from Carignan and Syrah (SYR1) and skins from Carignan and Alicante as being the most antioxidant ones.

## 3. Experimental Section

### 3.1. Experimental Materials

#### 3.1.1. Chemicals

Deionized water was purified with a Milli-Q water system (Millipore, Bedford, MA, USA). HPLC grade methanol and ethanol were purchased from Scharlau (Sentmenat, Barcelona, Spain). The following chemicals were obtained from Sigma Aldrich (St. Louis, MO, USA): (+)-catechin, (−)-epicatechin, B_1_ [(−)-epicatechin-(4β-8)-(+)-catechin], procyanidin dimer B_2_ [(−)-epicatechin-(4β-8)-(−)-epicatechin], cyanidin-3-*O*-glucoside chloride, delphinidin-3-*O*-glucoside chloride, malvidin-3-*O*-glucoside chloride, peonidin-3-*O*-glucoside chloride, gallic acid, 2,2-Diphenyl-1-picrylhydrazyl (DPPH), 6-hydroxy-2,5,7,8-tetramethylchroman-2-carboxylic acid (Trolox), 2,25,7,8-tetramethylchroman-2- carboxylic acid (Trolox) diammonium salt (ABTS), potassium persulfate, fluorescein, 2,2′-azobis (2-methylpropionamidine) dihydrochloride (AAPH), sodium dihydrogen phosphate dihydrate, disodium hydrogen phosphate dodecahydrate, 2,4,6-tri(2-pyridyl)-s-triazine (TPTZ), iron (III) chloride hexa-hydrate, iron (II) sulfate heptahydrate, Folin Ciocalteu’s phenol (2N), sodium bisulfite, sodium carbonate and formic acid. The Laboratory of Organic Chemistry and Organometallic (Université Bordeaux 1) synthesized procyanidin dimers B_3_ [(+)-catechin-(4α-8)-(+)-catechin], B_4_ [(+)-catechin- (4α-8)-(−)-epicatechin] and a trimer (C_1_) [(+)-catechin-(4β-8)-(+)-catechin-(4β-8)-(−)-epicatechin] [49].

#### 3.1.2. Plant Materials and Sample Preparations

In this study, samples were provided from Chateau Beaucastel, located in the Rhône Valley area. Grapes at maturity from the 2010 vintage underwent vinification processes after which their derived wines were used for commercial purpose. Grape pomaces were collected after these operations. Study was carried out using grape pomaces from *V. vinifera* L. cv. Grenache (from two different parcels [GRE1 and GRE2]), Syrah (from two different parcels [SYR1 and SYR2]), Carignan (CAR), Mourvèdre (MOU) and Alicante (ALI). Seeds were separated from skins using a mechanic separator and both were frozen at −20 °C prior to analysis. According to previous study ([50]), samples were selected on the basis of their high content of polyphenols. One hundred grams of GRE1, SYR1 and CAR seeds and GRE2, SYR1, SYR2, CAR, MOU and ALI skins were extracted in triplicate using 350 mL of distilled water for 1 h under magnetic agitation at 50 °C. In parallel, under the same conditions, these samples were also extracted using a 70% hydro/alcoholic (70:30, v/v) solution. The centrifugal supernatants were evaporated in vacuo at 30 °C and lyophilized to obtain two types of samples: an aqueous sample (EAQ) and a 70% hydro-alcoholic sample (EA70) for each variety and part.

### 3.2. Total Phenolics, Tannins and Anthocyanins

Total polyphenol, tannin and anthocyanin contents of grape pomace skin and seed extracts were determined. Crude extracts were solubilized in water/ethanol (90:10, v/v; pH 3.5 with tartaric acid) at appropriate concentrations. Total phenol content (TPC) was determined by the Folin-Ciocalteu assay [51] and the data expressed as mg of gallic acid equivalents (GAE) per g dry weight. Total tannin content was measured by acidic hydrolysis using the method of Ribereau-Gayon and Stonestreet [52]. Anthocyanin content was determined by the SO_2_ bleaching procedure [53].

### 3.3. HPLC Analysis of Monomeric/Oligomeric Tannins

Monomeric/oligomeric tannin extracts were solubilized in a methanol/water solution (50:50, v/v) at appropriate concentrations and analyses were carried out according to the method of Silva *et al.* 2011 [54]. 

### 3.4. HPLC Analysis of Anthocyanins

Grape pomace extracts were solubilized in methanol/water (50:50, v/v) containing 1% formic acid. Analysis were carried out using a Surveyor HPLC with sampler cooler maintained at 4 °C, a PDA detector scanning from 200 to 600 nm and a LCQ Advantage ion trap mass spectrometer with a split volume set at 0.2 mL/min and ESI operating in full-scan positive mode scanning from *m/z* 200 to 1000. Separation was performed on a 250 × 4.6 mm i.d. 4 µm Synergi RP-Max column (Phenomenex, Macclesfield, UK) maintained at 40 °C in a column oven. The mobile phase pumped at 1 mL/min comprised a 65 min, 10%–45% gradient of methanol in water with both solvents containing 1% formic acid. The injection volume was 10 µL. Peak detection and quantification were monitored at 520 nm and performed by comparison with available standards or confirmed by mass spectrometry in full-scan positive ionization, data dependent MS^2^. Anthocyanins were quantified as malvidin-3-*O*-glucoside equivalents and expressed in mg per dry weight of seed or skin ± SE (*n* = 3).

### 3.5. Antioxidant Assays

#### 3.5.1. Oxygen Radical Absorbance Capacity (ORAC) Assay

The ORAC assay was applied according to the method of Ou *et al.* [55] as modified by Dávalos *et al.* [56]. The procedure was carried out using an automated plate reader (BMG LABTECH, Ortenberg, Germany) equipped with a fluorescence detector set at excitation and emission wavelengths of 485 nm and 530 nm respectively. Analyses were conducted in a phosphate buffer (pH 7.4, 75 mM). Peroxyl radical were generated using AAPH (40 mM) and fluorescein (117 nM) was used as the substrate. Readings were taken every minute for 90 min at 37 °C. The area under the curve (AUC) was calculated for each sample by integrating the relative fluorescence curve. The net AUC was calculated by subtracting the AUC of the blank. The final ORAC values were determined by linear regression equation of Trolox concentrations and are expressed as µM Trolox equivalents/g dry weights.

#### 3.5.2. Ferric Reducing Antioxidant Potential Assay (FRAP)

FRAP assay was performed based on the method of Benzie and Strain [57] using an automated plate reader set at 593 nm. FRAP reagent were prepared daily by mixing 10 volumes of 300 mM soduim acetate buffer (pH 3.6) with 1 volume of 10 mM TPTZ solution and 1 volume 20 mM ferric chloride. A standard curve was prepared using various concentrations of FeSO_4_ × 7 H_2_O. Samples (40 µL) were allowed to react with FRAP reagent (300 µL) for 4 min in dark condition. Blank values were subtracted from samples and standards values then difference were used to calculate the FRAP value. Results were expressed as µM Fe^2+^/g of dry skin and seed weights.

#### 3.5.3. ABTS Assay

The ABTS assay was performed as described by Re *et al.* [58]. ABTS radical cation solution was prepared by mixing 2.45 mM of potassium persulfate and ABTS (7 mM in deionized water) following by 12–16 h incubation in the dark at room temperature. Before use, the ABTS^+•^ solution was diluted with deionized water to an absorbance of 0.7 ± 0.02 at 734 nm using a Jenway-6305 UV-vis spectrophotometer (Jenway, Staffordshire, UK). Samples (100 µL) were allowed to react with 2 mL of ABTS^+•^ solution for 10 min. Blank values were subtracted from samples and standard values and a linear regression for the Trolox standards were constructed. Results were expressed as µM Trolox equivalents/g dry weights.

#### 3.5.4. DPPH Assay

This method was used according to Brand-Williams *et al.* [59] modified by Miliauskas *et al.* [60]. Samples (100 µL) were allowed to react with 2 mL of daily prepared DPPH^•^ solution (6 × 10^−5^ M, dissolve in methanol) for 20 min at room temperature. The absorbance of the resulting solution was measured at 515 nm. Blank values were subtracted from samples and standard values. A linear regression for the Trolox standards was constructed. Results were expressed as µM Trolox equivalents/g dry weights.

### 3.6. Statistical Analysis

All measurements were performed in triplicate. Results are expressed as means ± standard deviation (SD). One-way ANOVA was performed to test the effects of variation factors (different samples) on each variable (TPC, total tannins, total anthocyanins, phenol concentrations *etc.*). If significant effects were found at a 95% confidence interval, ANOVA was followed by a Tukey’s HSD post hoc test to identify differences among groups. These analyses were performed using Statistica V.7 Software (Statsoft Inc., Tulsa, OK, USA).

## 4. Conclusions

This study showed that grape pomace seed and skin extracts still contained appreciable amounts of flavan-3-ols and anthocyanins, despite extraction during vinification. Overall, extraction with 70% aqueous ethanol was shown to optimize the recovery of flavan-3-ols and anthocyanins from the grape by-products. Indeed, by using a high alcohol level for the extraction of grape pomaces, less soluble and more stable compounds can be released while more soluble and less stable ones had already been extracted. Quantitative and qualitative distribution of polyphenols in grape pomaces showed significant differences across varieties. This investigation evidenced that seeds from Carignan and Syrah and skins from Carignan and Alicante as containing high phenolic contents and antioxidant activity. This information is of significant importance for the selection of suitable extracts which could be further used in cosmetics or anti-ageing products.
